# Case of Gonorrhœa Followed by Phagedœna Gangrœnosa, Hemorrhage, and Scaly Eruptions

**Published:** 1828-12

**Authors:** John Christopher Davie

**Affiliations:** Member of the Royal College of Surgeons, London.


### GONORRHŒA.

*Case of Gonorrhœa followed by Phagedæna Gangrænosa, Hemorrhage, and Scaly Eruptions.* By John Christopher Davie, Esq. Member of the Royal College of Surgeons, London.

A gentleman, æt. twenty-five years, residing near this place, consulted me, June 30th, respecting a gonorrhœa of a very severe kind. He had great difficulty and pain in voiding his urine, which was not passed oftener than once in twenty-four hours; considerable tenderness along the course of the urethra, and redness of the corona glandis, with an excessive discharge. These symptoms continued nearly three weeks, although leeches were applied to the perineum several times. The saturnine lotion was applied to the penis, the internal surface of the prepuce, and parts contiguous. Bowels acted upon freely by aperient medicine, state of quietude, and the antiphlogistic regimen strictly observed. Towards the 19th July, the continual pain had left him. He had still uneasiness when voiding his urine, and occasionally drops of blood passed. There were also distressing erections of the penis in the night, and the discharge from the urethra was of a more acrid nature, having a very offensive smell. For these symptoms the following was ordered·

℞. Copaibæ Balsami, Sp. Ætheris Nitrici, āā ℥i.; Tincturæ Opii ʒi. M. Capiat gtt. xl. ex cyatho aquæ ter die. Afterwards, powders of Cubeb, which somewhat ameliorated his sufferings.

July 26th.—Complained of a sore on the corona glandis, for which the following was had recourse to:

℞. Hydrargyri Submuriatis gr. xv.; Liquoris Calcis ℥ij. M. fiat lotio.

The sore at this time had not an unhealthy appearance. In the month of May he had three ulcers situated along the same tract, which yielded to the same application. He was strongly advised not to allow any of the discharge to get on the sore, which would be prevented by repeated washings with the former lotion, and the last application to the affected part.

27th.—The ulcer had a very foul appearance, and was somewhat below the surface of the skin. I touched it with lunar caustic.

28th.—The wound had a dark appearance all over its surface, more especially around its edges, somewhat extended, and attended with a burning pain. It was touched with the Potassa Fusa, which produced an eschar.

31st.—Slough separated, but the unhealthy aspect of the wound is not changed. Touched it freely with pure nitric acid.

August 3d.—This night felt considerably more pain than usual. Fifty drops of tincture of opium were given him. The slough had separated: one part of the sore had regained a healthy look, whilst the edges on the opposite side had a very dark appearance. The nitric acid was again applied upon lint, protecting the healthy part with dried lint.

7th.—Slough separated, and the unhealthy action of the wound is entirely changed, but it is not of a perfectly healthy granulating surface. There has been a slight discharge of blood, which I thought might have arisen from the separation of the dead parts from the living, as he used some force. In the middle of the sore there was a small black spot: it was dressed with a weak solution of nitric acid, five drops to an ounce of water.

8th, half-past five o'clock P.M.—Hemorrhage ensued to a most violent degree, bleeding through the dressings and rags wetted in cold water that were around it: prior to this it had bled three times in a slight degree, for which this morning it was dressed with Tinct. Benzoini c. Appetite not so good as it has been, and bowels constipated, probably from anodyne draughts taken at night to procure rest. Bowels relieved, Ol. Ricini ℥ss. He is of an extremely irritable habit and disposition, the slightest change putting him out of temper, and fancying that he never shall recover. A messenger was immediately despatched after me, and at a quarter-past six o'clock I arrived, and found him pale, languid, and with general coldness over his body, the parts still bleeding. Before this he had fainted. I immediately removed the dressings and the coagulated blood, in the hopes of finding the bleeding vessel: this however could not be effected, and, as he had lost a considerable quantity of blood (upwards of three pints in the whole), I used several dossils of lint to the part, and also around the penis. In about a quarter of an hour the bleeding ceased. Not the slightest movement of the body was permitted. Pulse tolerably good, considering the great quantity of blood lost. Port wine was occasionally given, and a nourishing diet directed.

11th.—There has been a considerable degree of fever, great thirst, heat of skin, white furred tongue; pulse between ninety and one hundred, full and strong; and bowels constipated. Wine discontinued, and low diet ordered. Salts and senna were taken to relieve the bowels. The parts below the lint are somewhat swollen and inflamed, having an erysipelatous blush. There has been a crackling heard on the opposite side of the wound, which discharges rather profusely, and has a disagreeable odour.—Dressings removed, and a considerable portion of the glans is absorbed; wound extending to the left side, but of a healthy nature. Dressed with Ceratum Album, taking care not to disturb the coagulum; rags wetted in cold water around; and perfect rest.

13th.—Coagulum separated.

16th.—The sore has a perfectly healthy aspect, discharging very profusely; the loose piece of the gland was brought together by adhesive plaster.

23d.—At half-past nine o'clock P.M. bleeding again ensued, but not in the same violent manner as before. The prepuce was swollen and inflamed, although lint had been applied to protect it. Dressings removed, and a finger applied to the part from whence the bleeding came, which was on the left side, next the corpora cavernosa, and it readily stopped. Dry lint used, and dressings of white cerate around the wound. Bowels constipated.

Ol. Ricini ℥ss. primo mane sumendus.

26th.—Has an unpleasant ulcer on the prepuce: it was dressed with Unguent. Hydrarg. et Nitrico Oxydi.

29th.—Has pustules generally about his face and body, and in the night felt great pain in the head of the tibia of the left leg.

September 2d.—The sore on the prepuce has somewhat extended, and its appearance very much resembles the one on the glans. Dressed with pure nitric acid. The pustules have terminated in scaly kind of eruptions, very much resembling those of syphilis, being of a coppery colour and elevated surfaces. Takes two pints of decoction of Sarsaparilla daily.

5th.—Slough somewhat lessened, and the edges of the wound look healthy. He was somewhat alarmed by the urine coming through the wound on the left side of the gland: this is the first time an unnatural aperture has been observed. His digestive powers are very good.

6th.—A flexible catheter was introduced, and allowed to remain to prevent the urine getting on the wound.

7th.—Slough separated, and wound healthy.

10th.—He expressed a particular wish for Mr. Ward, a professional friend, to see him with me, to which I did not object. At this time the loose piece of glans was quite healed, and that connected with the penis in part. A source of great irritation to the wound is the prepuce, which continually retracts over its surface, preventing its healing so readily as it would have done. The eruptions are nearly dispersed. He now took Quininæ Sulph. internally, and, where there was any appearance of a sore, stimulating ointments were applied. Catheter still used.

12th.—Catheter discontinued on account of being plugged up by mucus. No urine is observed to flow through the wound.

20th.—Eruptions entirely dispersed. Sarsaparilla and Quinine discontinued.

29th.—Still a small sore on the glans next the urethra. The urine passes through its natural aperture. There is still a thin pale discharge from that part, probably from the unhealthy state of the membrane lining the urinary canal. He has regained his strength.

When the first sore shewed a disposition to slough, I thought that it arose from the application of the virulent matter to the new skin of the wound that had been there before: since, I have been inclined to believe that constitutional causes had a great deal to do with it. He has been accustomed to live very freely, and has drank ardent spirits largely.

*College Green, Glocester.*

